# Prevention of Periprosthetic Joint Infection (PJI): A Clinical Practice Protocol in High-Risk Patients

**DOI:** 10.3390/tropicalmed5040186

**Published:** 2020-12-11

**Authors:** Ferdinando Iannotti, Paolo Prati, Andrea Fidanza, Raffaele Iorio, Andrea Ferretti, Daniel Pèrez Prieto, Nanne Kort, Bruno Violante, Gennaro Pipino, Alfredo Schiavone Panni, Michael Hirschmann, Marco Mugnaini, Pier Francesco Indelli

**Affiliations:** 1 Department of Surgical and Medical Sciences and Translational Medicine, Sapienza University of Rome, 00185 Rome, Italy; ferdinandoiannotti@gmail.com (F.I.); raffaeleiorio75@gmail.com (R.I.); aferretti51@virgilio.it (A.F.); 2 ASST Bergamo Ovest, 24047 Ospedale Treviglio, Italy; pprati60@gmail.com; 3 Department of Orthopaedic Surgery and Bioengineering, Stanford University School of Medicine, Stanford, CA 94305, USA; fidanza.and@gmail.com; 4 Department of Orthopaedic Surgery, Hospital del Mar, Universitat Autònoma de Barcelona, 08003 Barcelona, Spain; dr.danielperezprieto@gmail.com; 5 CortoClinics, 5482 Schijndel, The Netherlands; nanne@cortoclinics.com; 6 Orthopaedic Department, Istituto Clinico Sant’Ambrogio IRCCS Galeazzi, 20161 Milan, Italy; violante.b@gmail.com; 7 Ospedali Privati Riuniti Villa Regina, 40136 Bologna, Italy; dottgennaropipino@yahoo.com; 8 Department of Medical and Surgical Specialties and Dentistry, University of Campania “Luigi Vanvitelli”, 80138 Naples, Italy; Alfredo.SchiavonePanni@unicampania.it; 9 Department of Orthopaedic Surgery and Traumatology, Kantonsspital Baselland (Bruderholz, Liestal, Laufen), 4101 Bruderholz, Switzerland; michael.hirschmann@unibas.ch; 10 Division of Orthopaedic Surgery, Ospedale Santa Maria Annunziata Asl Toscana Centro, 50012 Firenze, Italy; marco.mugnaini@uslcentro.toscana.it

**Keywords:** TKA, PJI, periprosthetic joint infections, knee, hip, infection, prevention, DAPRI, musculoskeletal infections, local delivery, septic loosening

## Abstract

*Background:* Periprosthetic joint infection (PJI) represents 25% of failed total knee arthroplasties (TKA). The European Knee Associates (EKA) formed a transatlantic panel of experts to perform a literature review examining patient-related risk factors with the objective of producing perioperative recommendations in PJI high-risk patients. *Methods:* Multiple databases (Pubmed/MEDLINE, EMBASE, Scopus, Cochrane Library) and recommendations on TKA PJI prevention measures from the International Consensus Meetings on PJI from the AAOS and AAHKS were reviewed. This represents a Level IV study. *Results:* Strong evidence was found on poor glycemic control, obesity, malnutrition, and smoking being all associated with increased rates of PJI. In the preoperative period, patient optimization is key: BMI < 35, diet optimization, Hemoglobin A1c < 7.5, Fructosamine < 292 mmol/L, smoking cessation, and MRSA nasal screening all showed strong evidence on reducing PJI risk. Intraoperatively, a weight-based antibiotic prophylaxis, accurate fluid resuscitation, betadine and chlorhexidine dual skin preparation, diluted povidone iodine solution irrigation, tranexamic acid administration, and monofilament barbed triclosan-coated sutures for soft tissues closure all represented effective prevention measures. In the postoperative period, failure to reach normalization of ESR, CRP, D-dimer, and IL-6 six weeks postoperatively suggested early PJI. *Conclusion:* The current recommendations from this group of experts, based on published evidence, support risk stratification to identify high-risk patients requiring implementation of perioperative measures to reduce postoperative PJI.

## 1. Introduction

Periprosthetic joint infections (PJI) represent one of the worst complications following total joint arthroplasty (TJA) in general, and total knee arthroplasty (TKA) in particular. The clinical impact on patients is dramatic: the 5-year mortality rate following PJI equals the one of oncologic patients [1]. On the other side, hospital readmission rates following explants already double those of many cardiac and oncologic procedures [1], leading to a significant burden on healthcare systems. At current times, the combination of increasing antibiotic resistance and the growth in the number of culture-negative PJI [2] makes preventing infection a key aspect of adult reconstruction practices in order to avoid an epidemic escalation of PJI and musculoskeletal infections in general.

Unfortunately, effective prevention strategies to reduce the burden of this complication have not been fully determined. Decisions on patient selection and evaluation criteria, comorbidity detection, quantification of the perioperative risk, and application of countermeasures are often left on the shoulders of the treating physician, and not many standardized protocols for the prevention of PJI have been established [3].

This study aimed to review the current literature and multiple recommendations from well-recognized international scientific societies on preventive PJI measures with the objective to produce a clear, innovative, multimodal, perioperative protocol for TKA PJI prevention in high-risk patients. To achieve this goal, the European Knee Associates (EKA) formed a transatlantic panel of experts with a special interest in PJI and PJI prevention: the current authors first analyzed multiple modifiable patient-related and perioperative PJI risk factors, and secondarily produced guidelines for each of the three phases (preoperative, intraoperative, and postoperative) of the TKA procedure. The protocol presented here, despite representing the opinion of several EKA members, does not represent a consensus document from EKA. 

## 2. Preoperative Factors Increasing PJI Risk

### 2.1. Obesity

The American Academy of Orthopaedic Surgeons’ (AAOS) clinical practice guidelines [4] defined obesity as a moderate-strength criteria for increased risk. It has been described that patients with a body mass index (BMI) of 35 or greater have a two- to six-fold increased risk of PJI [5,6]. Consensus opinion from the American Association of Hip and Knee Surgeons (AAHKS) suggests that consideration should be given to delaying total joint arthroplasty in a patient with a BMI > 40, especially when associated with other comorbidities, such as poorly controlled diabetes or malnutrition [7].

### 2.2. Malnutrition

Since paradoxical malnutrition in patients having a high caloric but nutritionally poor diet is present in 42.9% of obese patients [8], malnutrition has been associated with a five to seven times greater risk of developing a major wound complication [9], ultimately leading to a PJI. Therefore, to cope with postoperative catabolic demands, nutritional supplementation has been strongly recommended to minimize PJI risk [10].

### 2.3. Diabetes Mellitus

Diabetes is a well-known risk factor for complications following TJA [11]. Historically, patients with uncontrolled diabetes have been found to have a 2.8 times increased risk of infection after TJA [12,13]. More recently, the critical role of acute glycemic control in patients undergoing TJA has been determined since multiple studies have shown patients with peri-operative hyperglycemia, not simply diabetes alone, have a significantly higher risk for complications [14,15]. Blood glucose levels between 110 and 180 mg/dL, a non-fasting glucose value less than 200 mg/dL, and a hemoglobin A1c value less than 7.5–8% have all been reported as ideal for elective TKA [13,14,15]. 

### 2.4. Smoking

Tobacco use and smoking are substantial risk factors for poor wound healing and infection. There is strong evidence that previous smokers had a similar risk profile to non-smokers: a few reports indicated that four weeks of cessation are required before elective surgery to attenuate the risk of surgical complications [16,17]. The normal value of serum cotinine assay has been reported as ≤ 10 ng/dl [18].

### 2.5. Skin Decolonization Prior to Surgery

Recent studies suggest the use of preoperative chlorhexidine cloth skin decolonization to reduce PJI after TJA because of its superiority compared to regular soap for preoperative cleansing of the skin—this has been demonstrated particularly in reducing infections related to MRSA [19,20]. The use of octenidine-palmitate (OL 11) has also been shown to reduce SSIs [21].

### 2.6. Nasal Decolonization

Since *S. aureus* nasal colonization correlates with increased risk for surgical site infections (SSIs) [22] and a few reports showed that an institutional decolonization protocol helped to decrease overall infection rate [23], the AAOS workgroup [4] suggested preoperative nasal mupirocin decolonization in all patients who are MRSA carriers because of its minimal potential risk of nasal irritation and its relatively low cost. 

## 3. Intraoperative and Perioperative Factors Increasing PJI Risk

### 3.1. Surgical Site Hair Removal

Kowalski et al. [24] recommended that hair removal should be considered in all patients undergoing elective joint arthroplasty, and should be performed by clippers before arrival in the operating room.

### 3.2. Perioperative Antibiotics 

Since the increasing number of MRSA and gram-negative PJIs [25], the classical single antibiotic prophylaxis prior to TJA has been recently challenged [26]. On the other side, Sewick et al. [27] demonstrated that the addition of vancomycin did not significantly reduce the rate of SSI when compared with cefazolin alone, but reduced the overall incidence of MRSA infections; because of this, it has been suggested by the same authors that only patients who are proven or potential carriers of MRSA, or those with a cephalosporin allergy, may benefit from vancomycin prophylaxis. This therapeutic strategy has also been shown to minimize the development of vancomycin-resistant *Enterococcus* [26,27].

### 3.3. Perioperative Antibiotics Timing

Although the Centers for Disease Control and Prevention (CDC) guidelines recommend a single preoperative dose in the case of TJA, there is a surprisingly limited amount of literature to support this recommendation [28,29]. It has been shown that a single perioperative dose of antibiotics does not increase the SSI/PJI rates if compared to multiple doses [28]. Interestingly, Inabathula et al. [30] reported that extended postoperative antibiotic prophylaxis up to seven days led to a statistically significant and clinically meaningful reduction in the 90-day infection rate in a selected group of high-risk patients. Claret et al. [31] also demonstrated a reduction in PJI when a prolonged post-operative antibiotic treatment was applied to total joint arthroplasty revisions. 

### 3.4. Surgical Site Skin Decolonization

Chlorhexidine-gluconate (CHG) has been recommended as the most efficient intra-operative surgical site preparation agent [10]; dual skin preparation, before and after draping and adding alcohol in a secondary scrubbing phase, has also shown favorable outcomes [20,32] as a preventive PJI measure.

### 3.5. Intraarticular Irrigation

Various intraoperative irrigation solutions have been recently studied. The World Health Organization (WHO), CDC, and International Consensus Meeting Clinical Practice Guidelines advocate for the use of diluted povidone-iodine (PID) irrigation during surgical procedures [29,33,34]; interestingly, cytotoxicity studies have shown that the bactericidal effect occurs even before individual human cells are affected [35]. Multiple studies have been performed in order to determine the optimal PID dilution in normal saline [36,37,38] prior to irrigation. Cichos et al. [38] evaluated the minimal inhibitory concentration (MIC) and time to bacterial death for 1% PID, 0.05% CHG, and 5 μg/mL vancomycin against multiple bacteria: those authors reported that PID, with a MIC of 0.63%, killed all tested micro-organisms immediately after contact, concluding that PID-accurate intra-articular diffusion was more important than extended exposure time. A recent in vitro study by Schmidt et al. [39] suggested that chlorhexidine may be a more effective irrigation solution for *S. epidermidis* eradication in biofilm than other commonly used solutions, such as povidone-iodine, Dakin’s solution, and triple antibiotic solution.

### 3.6. Fibrinolytic Agents

Since postoperative hematoma represents a well-known risk factor for PJI and SSI, the use of tranexamic acid (TXA) was recently introduced in many multimodal TKA protocols, since its use was strongly associated with reduced blood loss and decreased transfusion rates without an increase in thromboembolic complications [40]. 

### 3.7. Wound Closure

It has been reported that prolonged wound drainage (> 5 days) increases the risk of PJI by 13 times [41]: because of this, proper wound closing and postoperative wound monitoring represent key factors to avoid bacteria invading the joint space [41]. Recently, the use of a barbed monofilament suture was shown to provide a more watertight seal requiring no knots and allow for quick and cosmetic wound closure [42,43]. Interestingly, results from several meta-analysis studies showed that the incidence of SSI or wound infections decreased after using triclosan-coated sutures [44,45]. Since a moist environment protects the incision area from contamination, the use of silver-impregnated hydrofiber dressings have shown to decrease wound complications, the number of required re-dressings, and the rate of PJI by 4.6-fold [46,47]. In high-risk patients, wound checks and possible dressing changes should be performed on a daily basis—in the case of postoperative drainage, vacuum-assisted incisional dressing (iVAC) or negative-pressure wound therapy (NPWT) may play a role in reducing PJI risk [48]. 

### 3.8. Implant Surfaces

Since bacterial adhesion to the implant surface represents a key step of biofilm formation, several studies have focused on the relationship between prosthetic biomaterials and the increased risk of PJI [49,50,51]. Material and surface engineering led to the development of bactericidal/bacteriostatic modification of implant surfaces, such as chemical immobilization of antimicrobials, coatings with a broad range of antibacterial compounds (Gentamycin polymer, DAC hydrogel, silver-coated and iodine-coated implants), micro-textured surfaces, or anti-adhesion topographies: nevertheless, no method seems ideal, different results are reported, and no consensus exists on the use of a specific bactericidal surface [28]. 

### 3.9. Local Antibiotic Delivery

During an acute or chronic PJI, bacterial infiltration is mainly identified in the joint space which is poorly vascularized and more tolerant to bacteria proliferation before a local immune response can be stimulated. Because of this, a strategy to prevent bacterial colonization and biofilm formation may be needed to support a delayed and compromised immune response. Localized delivery of antibiotics is able to provide high concentrations of antibiotics that cannot be achieved systemically. Current evidence on the efficacy of Antibiotic-Impregnated Bone Cement (AIBC) in primary TKA is inconclusive [10,21,52]. Recently, calcium-sulphate antibiotic-added resorbable beads have received much attention, particularly due to their faster and longer elution compared to AIBC and poly-methyl-methacrylate (PMMA) beads. Unlike PMMA beads, calcium sulphate beads (CSB) do not need removal from the joint space and there is no risk of acting as a potential foreign body for bacterial colonization [53,54]. In vitro studies reported that calcium sulphate beads were capable of bacterial growth inhibition, preventing early bacterial colonization and biofilm formation by MRSA, *S. epidermidis,* and gram-negative bacteria by reaching localized antibiotic levels above the minimum inhibitory concentration (MIC) up to 39 days. However, various complications have been described when a higher volume of beads has been used—these include transient hypercalcemia, wound drainage, and heterotopic ossification [55,56].

## 4. Postoperative Factors increasing PJI Risk

PJI high-risk patients should be postoperatively followed-up using specific protocols, considering that fast-track programs with decreased length of stay (LOS) have shown a reduction in PJI when compared with standard protocols [20,57]. Few authors described the typical temporal patterns of CRP (C-reactive protein), ESR, IL-6 (interleukin 6), and D-Dimer in the early postoperative period [58,59,60,61,62,63,64]. It has been reported that the CRP value significantly increases and achieves its maximum between postoperative days 2 and 3 and, after this peak, it decreases to the normal level by the second postoperative week [25,44,48,65]. ESR usually elevates from postoperative day 1, peaks at postoperative day 5, and returns to the preoperative level between 12 and 26 weeks postoperatively [59,62,63]. The serum IL-6 is usually elevated on the first postoperative day, but falls to preoperative values at two weeks postoperatively [58,59,62]. D-dimer usually peaks at postoperative day 1, then decreases to nearly baseline level at day 2, and slowly elevates again, reaching the second peak at postoperative week 2 [61,64]. It has been shown that following TJA, a permanent elevation or abnormal patterns of these biomarkers could be effectively used in diagnosing early postoperative infection [60,61,64,66].

## 5. Protocol for PJI Prevention in High-Risk Patients

The current EKA panel of expert recommendations are based on strong evidence that preoperative patient optimization focused on the treatment of patient comorbidities could decrease the risk of PJI development.

### 5.1. Risk Calculator

Tan et al. [67] recently proposed an innovative, digital, PJI-relative risk calculator paradigm by determining the relative influence of demographic, surgical, and patient-specific factors in the development of a periprosthetic joint infection. While demographic factors included age, ethnicity, BMI, sex, age, and insurance, surgical factors included the joint location and a history of previous procedures, including being a primary or revision surgical intervention. Patient-specific factors included all major comorbidities. Cumulative point values and corresponding estimated PJI rates can be established on a case-by-case basis—this paradigm has been developed on the strong evidence that the most significant PJI risk factors are drug abuse, HIV positivity, presence of a major coagulopathy, renal disease, congestive heart failure, psychosis, rheumatologic disease, diabetes, malnutrition, liver disease, smoking, and high BMI. The current EKA panel of experts and current authors, adapting the risk calculator protocol [67], suggest to preoperatively stratify all patients undergoing TJA and to identify as high-risk patients those with an estimated PJI relative risk greater than 10% (130 points). Here, the current authors propose a preventive protocol originally designed for PJI high-risk patients, but is expandable to all TJA patients, implementing preoperative, intraoperative, and postoperative measures.

#### 5.1.1. Preoperative Measures 

In the preoperative setting, patient education and optimization are key factors (Table 1). A pre-hospitalization visit should be performed at least 30 days before surgery with appropriate specialist consultations in order to optimize the treatment of comorbidities when this is possible. 

The current panel of authors recommend that patients with a BMI > 35 and who are at high PJI risk, and patients with a BMI > 40 and at medium or low PJI risk, should have their TJA delayed and optimized by undergoing nutritional and bariatric surgery consultations [4,7,68,69]; in the case of persistently high BMI and disabling and painful osteoarthritis, intraoperative prevention measures are key. 

Patients with poor preoperative nutritional status should have their diets optimized, aiming to obtain lymphocyte count >1500 cells/mL, albumin value >3.5 g/dL, zinc levels >5 mg/dL, and transferrin value > 200 mg/dL [8,9,10]. Regarding hyperglycemia and diabetes, TJA should be delayed until the following preoperative serologic values are reached: fasting glucose level < 180 mg/dl, not-fasting glucose < 200 mg/dL, Hemoglobin A1c < 7.5%, and Fructosamine < 292 mmol/L [13,14]. If the patient is a smoker, the current authors recommend smoking cessation at least four weeks preoperatively and a serum cotinine assay ≤ 10 ng/dl at the time of surgery [16,17]. Furthermore, we suggest screening for nasal MRSA and to treat positive patients with nasal Mupirocin twice a day for five days preoperatively [70]. Another recommendation in high-risk patients is to take a bath with chlorhexidine-based soap/solution, or to use CHG cloths the night before and the morning of surgery, and have the patient to sleep in clean garments and bedding during the preoperative night.

In patients at high risk for MRSA PJI (positive preoperative nasal colonization, nursing-home residents and health-care workers) we recommend they take a bath with chlorhexidine daily for five days before surgery (Table 1). Skin preparation before surgery should be done at home by shaving with electric clippers.

#### 5.1.2. Intraoperative Measures

Peri-operative antibiotic prophylaxis is crucial in patients at high risk for PJI. In this scenario, the current panel of authors do not recommend a single prophylactic agent. A weight-based gentamicin prophylaxis could be added to the traditional cephalosporin prophylaxis, dosing it on the basis of the patient’s ideal body weight plus 40% of the difference between the actual and ideal body weight [28]. It has been recommended that cefazolin infusion should end 30 to 60 min prior to the skin incision, and the standard preoperative dose (1 g of cefazolin) should be doubled in patients with a weight > 80 kg, and tripled if > 120 kg. The current authors recommend, in the case of a large intraoperative blood volume loss (> 1500 cc), a high volume of fluid resuscitation (> 1500 cc), and surgery length reaching more than the half-life of the prophylactic agent (or longer than 3–4 h), to add an additional intraoperative dose of antibiotics [28,71]. 

In MRSA-risk patients or in institutions with a high prevalence of MRSA (>20%), we recommend the use of vancomycin prophylaxis at a dose of 15–20 mg/kg in addition to the standard antibiotic (Table 1) [71,72,73]. There is recent evidence [29] which suggests prolonging the oral antibiotic prophylaxis in high-risk patients for at least a week postoperatively. 

At the first author’s institution, all high-risk patients undergo preoperative betadine and chlorhexidine skin preparation before and after draping, followed by the use of surgical adhesive incise drapes with bacteriostatic agents [32]; irrigation with 0.65% diluted povidone iodine solution is then performed before and after final implant positioning by mixing 30 mL of sterile 10% povidone-iodine with 500 mL of 0.9% saline. The current literature suggests that the diluted povidone iodine solution does not need to be left in the joint for 3 min as previously published [29,33,34], but as long as required to thoroughly expose it to all areas of the joint. After suction, a pulsatile lavage with 1 L of normal saline is performed.

The current authors, following a recent systematic review by Abosala et al. [74], recommend the intra-articular use of calcium sulphate antibiotic-impregnated beads (Stimulan; Biocomposites Ltd., United Kingdom) in patients considered at high risk for a periprosthetic joint infection—in those cases, a 10 mL kit of PG-CSH is routinely mixed with 1000 mg of vancomycin hydrochloride powder and 6ml of a 40 mg/mL tobramycin solution or 320 mg of gentamycin. A smooth paste is ultimately formed by mixing all components for 60 s and is pressed into 4.8 mm diameter hemispherical cavities in a flexible mold.

At the senior author’s institution (PFI), tranexamic acid is routinely administered both topically (1 g intraoperative), as well as intravenously (1 g preoperatively, 1 g after 3 h, and 1gr after 6 h from the TJA procedure); we also follow the recommendations of Wu et al. [45] on using monofilament barbed triclosan-coated sutures, since barbed subcuticular sutures with surface glue application have been proved to be an excellent option on reducing the incidence of prolonged wound discharge. An occlusive hydrofiber silver impregnated dressing can also be applied on the wound and kept in place for at least 48 h.

#### 5.1.3. Postoperative Measures

Fast-track programs should aim for earlier discharge, as previously recommended by several EKA members [75]. However, in the early postoperative period, attention must to be paid to glycemic control, urinary infection management, and wound drainage [70]. The current authors here propose a postoperative protocol for high-risk patients—this protocol focuses on monitoring several serological biomarkers, and on wound drainage, multiple checks and management (Table 2). Wound dressings should be monitored and changed in the first 48 h postoperatively, only if more than 50% of dressing saturation is present; if drainage persists for more than 72 h, the current authors strongly recommend using iVAC or NPWT dressings. The patient should be closely monitored, and if the wound persistently drains for more than 7 days postoperatively, irrigation and debridement should be considered without delay [48,76]. 

Elevated values or abnormal patterns of ESR, CRP, D- dimer, and IL-6 test following TJA should be effectively used in diagnosing early postoperative infection—a suspicion of developing an acute PJI should be raised when there is no decrease of D-dimer in the second postoperative day, no decrease of CRP and IL-6 at two weeks, and no normalization of all serologic markers at six weeks from surgery. In such a scenario, a synovial fluid analysis (cell count and differential, culture) becomes mandatory. A recent and very promising technique for microorganism isolation in this scenario is represented by cell-free DNA Next Generation Sequencing [77]. The current authors strongly recommend prompting irrigation and debridement or a DAPRI procedure [76] in order to preserve the implant before the establishment of drug-resistant biofilm on the implant, or before osteomyelitis becomes entrenched in the periprosthetic bone.

## 6. Conclusions

Prevention is a key factor to reduce the rate of PJI, and surgeons should use multiple, evidence-based measures at each step of the surgical process to stop the possible development of this complication. The current panel of authors support risk stratification in order to make possible to identify high-risk patients who require targeted measures, ranging from patient education and rigorous preoperative optimization to perioperative additional measures and close monitoring in the postoperative period.

## Figures and Tables

**Table 1:** Preventive Measures in High-Risk Patients (>130 pts) with Additional Methicillin-Resistant Staphylococcus aureus (MRSA) Risk.

MRSA RISK	Measures	
1. MRSA Risk patients Nasal colonization + Nursing home residents Health-care workers 2. Institutions with a high prevalence of MRSA	Nasal decolonization	Mupirocin twice a day for 5 days
	Skin decolonization	Bath with chlorhexidine daily for 5 days before surgery
	Antibiotic prophylaxis	Vancomycin prophylaxis at a dose of 15−20 mg/kg in addiction to the standard antibiotic
	Local Antibiotic Delivery	Calcium sulphate beads with vancomycin

**Table 2:** Postoperative Monitoring Protocol in High-Risk Patients (> 130 pts).

Postoperative	Serological Test	Wound Management	
Day 1	ESR, CRP, D- dimer, IL-6	Check drainage	
Day 2–3	ESR, CRP, D- dimer, IL-6	< 50% dressing saturation→ Monitoring	> 50% dressing saturation→ Change dressing
Day 3–5	Discharge		Persistent Drainage→iVAC
Days 5–7		Wound dressing change	Persistent Drainage →DAIR/DAPRI
Days 14	High CRP, IL-6 → repeat at 4 weeks	Suture removal or wound check (if glue)	
Week 4	High CRP, IL-6 → consider synovial fluid analysis		
Week 6	No Normal value of ESR, CRP, D- dimer→ synovial fluid analysis →DAIR/DAPRI		

## Data Availability

All underlying data are in the text and tables.
